# A Method for Autonomous Multi-Motion Modes Recognition and Navigation Optimization for Indoor Pedestrian

**DOI:** 10.3390/s22135022

**Published:** 2022-07-03

**Authors:** Zhengchun Wang, Zhi Xiong, Li Xing, Yiming Ding, Yinshou Sun

**Affiliations:** 1 College of Automation Engineering, Nanjing University of Aeronautics and Astronautics, Nanjing 210016, China; wangzc@nuaa.edu.cn (Z.W.); dingyiming@nuaa.edu.cn (Y.D.); sunyinshou@nuaa.edu.cn (Y.S.); 2 Navigation Research Center, School of Automation Engineering, Nanjing University of Aeronautics and Astronautics, Nanjing 211106, China; 3 School of Railway Transportation, Shanghai Institute of Technology, Shanghai 201418, China; xl@sit.edu.cn or

**Keywords:** multi-node inertial sensor network, multi-motion modes recognition, zero-velocity detection, heading correction, pedestrian navigation

## Abstract

The indoor navigation method shows great application prospects that is based on a wearable foot-mounted inertial measurement unit and a zero-velocity update principle. Traditional navigation methods mainly support two-dimensional stable motion modes such as walking; special tasks such as rescue and disaster relief, medical search and rescue, in addition to normal walking, are usually accompanied by running, going upstairs, going downstairs and other motion modes, which will greatly affect the dynamic performance of the traditional zero-velocity update algorithm. Based on a wearable multi-node inertial sensor network, this paper presents a method of multi-motion modes recognition for indoor pedestrians based on gait segmentation and a long short-term memory artificial neural network, which improves the accuracy of multi-motion modes recognition. In view of the short effective interval of zero-velocity updates in motion modes with fast speeds such as running, different zero-velocity update detection algorithms and integrated navigation methods based on change of waist/foot headings are designed. The experimental results show that the overall recognition rate of the proposed method is 96.77%, and the navigation error is 1.26% of the total distance of the proposed method, which has good application prospects.

## 1. Introduction

In recent years, with the development of society, pedestrian navigation technology has received more and more attention and research from researchers in the fields of disaster relief and rescue, medical search and rescue, public safety, fitness consumption, counterterrorism, and so on. Traditional pedestrian navigation and positioning methods mainly rely on the global navigation satellite system (GNSS) [1]. In real life, pedestrians are not always in an environment with good GNSS signals. When pedestrians are in buildings, on viaducts, in forests with poor GNSS signals, or even in underground parking lots, indoor, tunnels and other environments without satellite signals, GNSS will not be able to provide accurate navigation and positioning functions for pedestrians. In addition, the indoor motion modes of pedestrians are not only limited to walking, but also common motion modes such as running, going upstairs, and going downstairs. Especially in a special application environment such as during a rescue, it is necessary to monitor the movement status of special operators in real time for the command and dispatching center to perceive their motion mode and trajectory. It is convenient for decision makers to adjust task strategies in time according to their own conditions and the positions of operators. Therefore, there is an urgent need to carry out research on autonomous recognition and navigation optimization methods for multi-motion modes for indoor pedestrians in the satellite failure environment to meet the needs of practical applications.

At present, indoor pedestrian navigation technology mainly includes active navigation algorithms and passive navigation algorithms. Active algorithms mainly include using ultra-wide band (UWB) [2], Wi-Fi [3,4], visual sensor [5], Bluetooth, ZigBee, radio frequency identification (RFID) and other multi-source information for fusion positioning. The navigation and positioning errors of this information do not accumulate with the passage of time, and the accuracy is also high. A passive navigation algorithm is a pedestrian navigation method that only uses IMU. This kind of method does not need to set up reference facilities in advance, nor does it need to send signals to the outside world. It only needs to fix the IMU on feet, legs, waist, and other parts of the human body, and conduct pedestrian navigation and positioning by collecting IMU data. IMU has the advantages of strong autonomy, convenient installation, and low cost. However, the accuracy of IMU is limited, and the navigation error will diverge with time.

There are two common pedestrian passive navigation methods based on IMU. One is the pedestrian dead reckoning (PDR) technology, which calculates step length and heading of a pedestrian through acceleration and angular velocity and calculates pedestrian position; The second method is the strapdown inertial navigation algorithm based on zero-velocity update (ZUPT). Many people have carried out research on pedestrian navigation algorithms based on PDR algorithm. In 2019, Xu l [6] proposed a PDR navigation algorithm based on handheld mobile phones, which improves navigation accuracy through a zero angular velocity heading correction algorithm and lateral velocity constraint algorithm. In 2020, Ding, Y. [7] proposed a navigation algorithm based on three node IMUs of legs and waist, and built an inertial map on the basis of PDR for secondary correction. However, the PDR algorithm needs to estimate the accurate step and heading to provide accurate navigation and positioning services for pedestrians. 

So, many people have carried out research on pedestrian navigation algorithms based on a ZUPT algorithm. Eric Foxlin [8] first studied the pedestrian navigation technology based on ZUPT of foot, which can effectively suppress the error divergence of inertial navigation system; In 2016, Tian, X. [9] et al. studied and established the functional relationship between the zero-velocity detection threshold and the step frequency, which can accurately recognize the zero-velocity interval of walking under different step frequencies; In 2020, Jesus D. Ceron et al. [10] proposed a foot-mounted navigation and positioning algorithm for walking, running and jogging. The positioning accuracy of the algorithm using complementary filtering is 8.8%; In 2021, Li, W. [11] studied a ZUPT navigation algorithm based on the constraint of biped maximum distance inequality. The algorithm was applicable to walking, running and their mixed movements. The pedestrian’s position and posture are calculated through acceleration, angular velocity, magnetic intensity, air pressure and other data. The navigation parameters are modified by using the zero-velocity information of foot during walking. In the research of passive pedestrian navigation based on IMU, course correction is always a problem to be solved; In 2015, Xu, Y. et al. [12] studied the pedestrian navigation method based on dual IMUs on shoulder and foot, which corrected the heading of the navigation system through the heading difference between shoulder and foot; In 2015, Min, L. et al. [13] studied the navigation algorithm of heading of waist and magnetic data to assist feet; In 2018, Wang, H. et al. [14] studied the recognition of straight line and turning motion, extracted the true heading from the accelerometer and magnetometer, and reduced the heading deviation in straight-line motion; In 2019, Shi, L. F. et al. [15] proposed an adaptive error estimation method of IMU and a heading correction method using geomagnetic correction. Using low-precision IMU can also maintain a certain positioning accuracy. The ZUPT method proposed in that paper is suitable for running. Because there are many indoor magnetic interferences, the effect of using magnetism to correct indoor pedestrian navigation heading will be greatly reduced; In 2019, Niu, X. [16] et al. proposed an equality constraint method based on pedestrian track, which constrained navigation information through biped distance. But it only studied the walking state. For the indoor pedestrian navigation algorithm based on ZUPT, two-dimensional navigation is mainly studied. At the same time, researchers have carried out some research on different installation parts of IMU, but there are few research studies on navigation in common mixed motion modes. Some algorithms use geomagnetic data for heading correction, which is susceptible to interference.

There are many common modes of movement for indoor pedestrians. Accurate detection of pedestrian motion modes is an important basis for improving the accuracy of indoor navigation. Different motion modes use different zero-velocity detection algorithms. Pedestrian motion mode recognition methods mainly include traditional threshold method and the machine learning method. The parameters of traditional threshold method are fixed values. Even in the same motion mode, the motion parameters of pedestrians are not exactly the same. Therefore, using the threshold method [17] to identify the modal adaptability is insufficient; In the machine learning classification method, the machine learning model is trained according to the data eigenvalues of different motion modes to realize pedestrian motion modes recognition. Common pedestrian modal recognition methods with machine learning include support vector machine (SVM) [18,19], k-nearest neighbor (KNN), artificial neural networks (ANN) [20], decision tree (DT) [21], etc. The conventional SVM algorithm has high accuracy for binary classification. For many types of classification, there are problems of reduced calculating speed and accuracy; KNN algorithm needs to set the K value in advance and balance the number of various samples; ANN training models need a large number of parameters, take a long time to learn, and even have the possibility of unsuccessful learning. The DT algorithm has the problem of over fitting. In addition, different mounting positions have different effects on different motion mode recognition. Since 2015, the German Aerospace Center (DLR) has carried out research on running, climbing, lying and other motion modes recognition with multi IMUs [22], and found that an inertial measurement unit (IMU) worn on leg has a good effect in gait segmentation research. In 2016, Liu, Y. [17] et al. fixed the IMU at the waist and studied a classification method based on the time domain eigenvalue threshold method, which can recognize 8 daily motion modes such as static, walking, running, and jumping. In 2017, Wagstaff, B. [18] et al. studied a motion classification method based on a single IMU sensor mounted on foot. The method recognized six motion modes of walking, jogging, running, sprinting, crouching walking and climbing stairs with SVM, and studied the navigation and positioning error algorithm for walking and running. In addition, literatures [19,20,21,23] also studied the motion mode recognition algorithm based on handheld IMU, respectively, and obtained useful conclusions. Since the threshold value in the threshold method is fixed, and the machine learning method is more tolerant, so the machine learning method is selected to recognize pedestrian motion modes. Pedestrian motion has time-series characteristics, but general machine learning methods are not sensitive to time-series characteristics. When a machine learning algorithm is used to recognize motion modes, appropriate eigenvalues of motion should be selected.

In order to effectively solve the needs of autonomous navigation for special tasks such as emergency rescue and disaster relief, medical search and rescue, a multi-motion mode pedestrian navigation method based on multi-inertial sensors is proposed for indoor special operators with multiple motion modes. In this paper, an inertial sensor network based on four-node inertial sensors of waist, legs and right foot is designed. According to the characteristics of four common pedestrian motion modes of indoor walking, running, going upstairs, and going downstairs, the feature values of the legs are extracted to segment each step of the movement. The appropriate eigenvalues are selected for each step, and the four motion modes are recognized by Long short-term memory (LSTM). In view of the short zero-velocity interval in the running mode, a set of zero-velocity detection methods cannot be simply used for the four motion modes. The motion modes are divided into two categories: walking, going upstairs, and going downstairs as one category, and running as one category. Each type of motion uses a set of zero-velocity detection algorithms; For passive navigation, the heading of foot inertial navigation system based on ZUPT still diverges. At the beginning and end of a step, the change of the waist heading, and the change of the foot heading should both be 0°. The difference between the change of the waist heading and the change of the foot heading is constructed as a virtual observation to assist in the heading correction of pedestrians. The experimental results show that the method proposed in this paper can effectively recognize the motion modes of normal walking, running, going upstairs, and going downstairs. The proposed navigation optimization method based on waist/foot angular velocity to assist in correcting heading errors can meet the needs of autonomous navigation for special tasks.

The following sections of the paper are organized as follows: The general framework of the algorithm is introduced in Section 2; Section 3 introduces the autonomous method of multi-motion modes recognition for indoor pedestrians; Section 4 describes the ZUPT/waist/foot navigation optimization algorithm model; In Section 5, the experimental results are systematically analyzed. Section 6 presents a discussion of the method.

## 2. Navigation Scheme Based on Multi-Node Inertial Sensor Network

**Figure 1:**
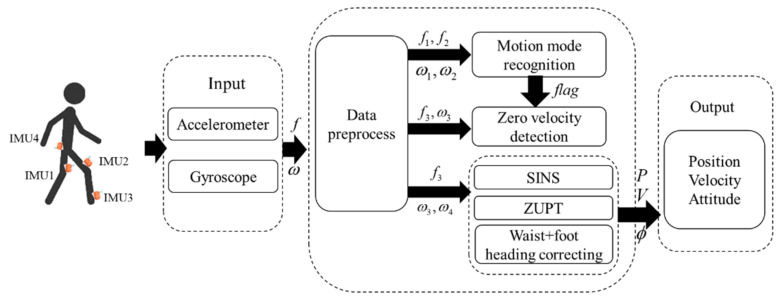
Four-node inertial sensor network and pedestrian navigation system.

The traditional motion mode recognition system uses the fixed threshold method to recognize motion modes, and the recognition rate is high when the motion velocity is close. However, when the pedestrian motion changes, there will be missed detection and false detection in motion mode recognition; In addition, the traditional indoor ZUPT algorithm can only correct the position and velocity information of pedestrians. Without appropriate observable information to correct the heading, it will diverge after a long time, which will lead to excessive position error. Generally, IMUs are mainly installed on foot, leg, waist, shoulder, and other parts. Because the motion characteristics of various parts of the human body are different, IMUs installed on different positions of a human body have different advantages. Therefore, according to the characteristics of different parts of the human body and different motion modes, the advantages of IMUs on different parts of the human body are fully utilized to overcome the shortcomings of the traditional fixed threshold method for recognizing pedestrian motion modes, as well as the problem of no heading observation for indoor pedestrian. A navigation implementation scheme based on a four-node inertial sensor network is proposed. The designed four-node inertial sensor network framework is shown in Figure 1.

The four nodes of the inertial sensor network are, respectively, arranged on the right foot/left leg/right leg/waist. The pedestrian four-node inertial sensing network will be divided into two sub-inertial systems: the two IMUs installed on the legs are used as a sub-inertial system 1, and the two IMUs installed on the right foot and waist are used as the sub-inertial system 2. In Figure 1, IMU1, IMU2, IMU3, and IMU4 represent inertial sensors installed on left leg, right leg, right foot, and waist, respectively. $f_{1}$, $f_{2}$, $f_{3}$, $f_{4}$, $\omega_{1}$, $\omega_{2}$, $\omega_{3}$, $\omega_{4}$ are the acceleration and angular velocity of inertial sensors IMU1, IMU2, IMU3, and IMU4, respectively. The order of *x*, *y*, and *z* axes of the body coordinate system of the sensor is right, forward, and upward, which satisfies the right-hand rule. The navigation coordinate system is east, north, and universe (ENU).

The algorithm proposed in this paper is as follows: First, the Euclidean Distance is used to identify static and dynamic modes; For dynamic modes, the inertial data of legs are used for gait segmentation, and the motion mode is recognized by the LSTM algorithm based on the motion features of each step; Then, according to different motion modes, zero-velocity interval of foot is adaptively detected; Finally, the pedestrian navigation system is corrected according to ZUPT and difference between waist/foot heading of a foot of each step.

## 3. Autonomous Method of Pedestrian Indoor Multi-Motion Modes Recognition

### 3.1. Definition of Multi-Motion Modes

**Figure 2:**
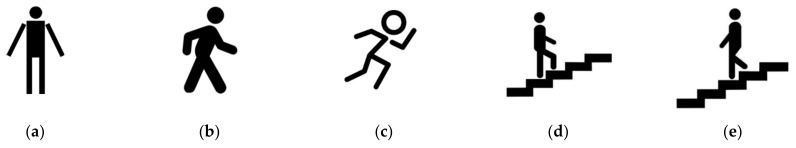
Schematic diagram of multi-motion modes. (**a**) Static; (**b**) Walking; (**c**) Running; (**d**) Going upstairs; (**e**) Going downstairs.

Indoor multi-motion modes of pedestrians are defined as shown in Figure 2. (a) Static: stand upright and perpendicular to the ground, keep your feet close to the ground and do not shake your body; (b) Walking: the body moves forward at a low speed; (c) Running: the body moves forward at a fast speed; (d) Going upstairs: walk up the stairs; (e) Going downstairs: walk down the stairs.

### 3.2. An Autonomous Method for Multi-Motion Modes Recognition

For motion mode recognition, traditional methods employ windowing studies for each data point. When there are many motion modes to be recognized, the recognition accuracy is insufficient.

**Figure 3:**
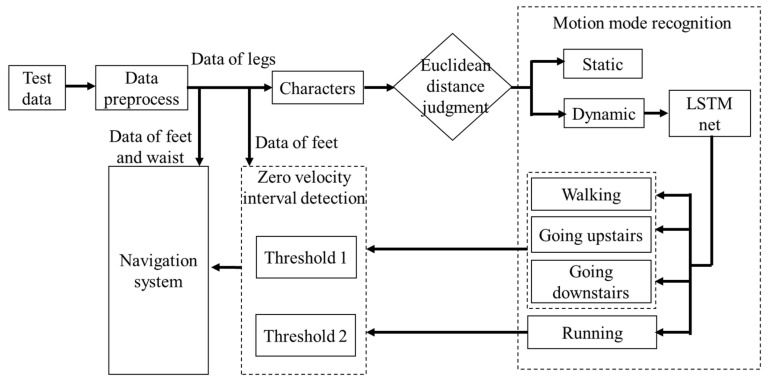
Scheme of the autonomous method for multi-motion modes recognition and zero-velocity detection for indoor pedestrian.

For the four motion modes studied in this paper: walking, running, going upstairs and going downstairs, it is necessary to use the characteristics of different installation positions of IMUs to realize recognization. During the movement, leg movement has strong regularity and attitude angle changes are relatively stable, so leg angular velocity can be used for motion mode recognition. The motion mode recognition method is to first roughly divide the pedestrian motion modes into static and dynamic, and then subdivide the four modes under motion modes. Since running is relatively faster than walking, going upstairs, and going downstairs, and the zero-velocity interval of running of feet is short, the motion modes can be divided into two categories: walking, going upstairs and going downstairs as steady motion, and running as non-steady motion. The two categories of motion modes adopt different zero-velocity detection methods. The overall algorithm scheme of mode recognition is shown in Figure 3.

There are two data selection methods for motion mode recognition, the first is based on each data point, and the second is based on the data within each step. The advantage of the first recognition method is that it is simple and fast, but the eigenvalues extracted from each moment may appear in several motion modes or several states have similar eigenvalues, so this method has too many limitations. The second recognition method is based on the inertial sensor information characteristics of various gaits. Firstly, each step in the moving process is accurately segmented to provide the basis for the eigenvalues of each step, and then the threshold method or machine learning method is used to classify the motion modes. This paper adopts the second method, which is based on gait segmentation for pedestrian motion mode recognition.

#### 3.2.1. Data Preprocessing Algorithm

(1) Projection of gravity component

Due to the installation method of the IMU, the three axes *x*, *y*, and *z* of the IMU body coordinate system do not coincide with the east, north, and universe directions of the navigation coordinate system, and the gravity has components in the three axes. Before the motion mode recognition, the components of gravity in the three axes are deducted, and the obtained horizontal acceleration and vertical acceleration data can better reflect the motion characteristics of pedestrians. The acceleration measurement data saved by the IMU is denoted as $a=\left(f_{x},f_{y},f_{z}\right)$. The mean value of the static three-axis acceleration data is denoted as $\bar{a}=\left({\bar{f}}_{x},{\bar{f}}_{y},{\bar{f}}_{z}\right)$. The linear acceleration minus the gravitational component is denoted as $a_{l}$.

(1) $$a_{l}=a-\bar{a}$$

Decompose $a_{l}$ into vertical and horizontal directions. The vertical projection of the linear acceleration $a_{l}$ on the reference gravity vector is denoted as $a_{v}$.

(2) $$a_{v}=\left(\frac{a_{l}\cdot a}{a\cdot a}\right)\cdot a$$

The horizontal projection of linear acceleration $a_{l}$ with respect to the reference gravity vector $\bar{a}$ is denoted by $a_{h}$. $a_{h}$ is obtained by vector subtraction, which represents the component of acceleration on the horizontal plane.

(3) $$a_{h}=a_{l}-a_{v}$$

The data of leg acceleration minus gravity component is denoted as $a^{\prime }=(a_{vx},a_{vy},a_{h})$,

(2) Filter algorithm

In the process of pedestrian movement, the leg changes are periodic. The periodicity is mainly reflected in the acceleration and angular velocity of IMU. The raw data collected by IMU contains a lot of noise and errors, mainly including two types of sources: one is the noise of the IMU itself, and the other is the noise caused by the unstable movement of the experimenter. For angular velocity, it needs to be smoothed and denoised before gait segmentation. In this paper, a Savitzky–Golay (SG) filter is used for data preprocessing, which is a polynomial smoothing algorithm based on the local polynomial least squares principle in the time domain. The SG filter has the advantage of removing high-frequency noise while keeping the shape and width of the original signal unchanged. The basic principle of the SG filter is as follows:

The length of the window is set as n=2m+1. The n measuring points within the window are shown in Equation (4), and the measuring points are in sequence

(4) $$x=\left(-m,-m+1,\ldots -1,0,1,\ldots ,m-1,m\right)$$

The least squares fitting is performed on the data in the window using k−1 degree polynomial, as shown in the following Equation (5):

(5) $$y=a_{0}+a_{1}x+a_{2}x^{2}+\cdots +a_{k-1}x^{k-1}$$

In the equation, x is the variable, $a_{i}$ is the polynomial coefficient, and y is the central point data.

Using Equation (5), n equations are established, forming a k element linear equation system, which is represented by a matrix as Y=XA+ε. Where Y is the window data vector, X is the independent variable vector, A is the polynomial coefficient matrix, ε is the residual vector, The following equation holds:

(6) $$\left[\begin{array}{c} y_{1}\\ \vdots \\ y_{m-1}\\ y_{m}\\ y_{m+1}\\ \vdots \\ y_{n} \end{array}\right]=\left[\begin{array}{ccccc} 1 & x & x^{2} & \cdots & x^{k-1}\\ \vdots & \vdots & \vdots & \cdots & \vdots \\ 1 & x & x^{2} & \cdots & x^{k-1}\\ 1 & x & x^{2} & \cdots & x^{k-1}\\ 1 & x & x^{2} & \cdots & x^{k-1}\\ \vdots & \vdots & \vdots & \cdots & \vdots \\ 1 & x & x^{2} & \cdots & x^{k-1} \end{array}\right]\left[\begin{array}{c} a_{0}\\ a_{1}\\ a_{2}\\ \vdots \\ a_{k-1} \end{array}\right]+\left[\begin{array}{c} \varepsilon_{1}\\ \vdots \\ \varepsilon_{m-1}\\ \varepsilon_{m}\\ \varepsilon_{m+1}\\ \vdots \\ \varepsilon_{n} \end{array}\right]$$

In order to have a solution to the system of equations, n≥k is required, and n>k is generally selected. The polynomial coefficient matrix $\hat{A}$ and the filter value $\hat{Y}$ are determined by least squares fitting, and the calculation equation is as follows:

(7) $$\hat{A}={\left(X^{T}\cdot X\right)}^{-1}\cdot X^{T}\cdot Y$$

(8) $$\hat{Y}=X\cdot \hat{A}=X\cdot {\left(X^{T}\cdot X\right)}^{-1}\cdot X^{T}\cdot Y$$

In the process of SG filter, it is found that for window size n, the larger n is, the more effective signals are lost. For the fitting order k, the larger k is, the worse the denoising ability is.

(3) Zero Bias Compensation of Gyroscope

Due to the characteristics of IMU, the gyroscope has drift error. In this paper, the method of deducting zero deviation is used to reduce the drift error of gyroscope. Relative to the experimental time, it is considered that the zero bias of the gyroscope remains unchanged during the experimental time. Gyroscope zero bias is calculated using angular velocity during a period of static time before navigation begins. After the navigation data is updated at each moment, the zero offset is deducted from the angular velocity of the IMU. Angular velocity measurement data saved by the IMU is denoted as $\omega =\left(\omega_{x},\omega_{y},\omega_{z}\right)$; The mean value of the three-axis angular velocity data in the static interval, that is, the angular velocity zero offset, is recorded as $\bar{\omega }=\left({\bar{\omega }}_{x},{\bar{\omega }}_{y},{\bar{\omega }}_{z}\right)$; The angular velocity after deducting the zero offset is $\tilde{\omega }$.

(9) $$\tilde{\omega }=\omega -\bar{\omega }$$

#### 3.2.2. Recognition Algorithms for Dynamic and Static Mode

In the process of pedestrian movement, the difference of the pitch angle of both legs is periodic, which is reflected in the difference of the right angular velocity of the two legs.

(10) $$\Delta \omega_{leg}=\omega_{ll}-\omega_{rl}$$

**Figure 4:**
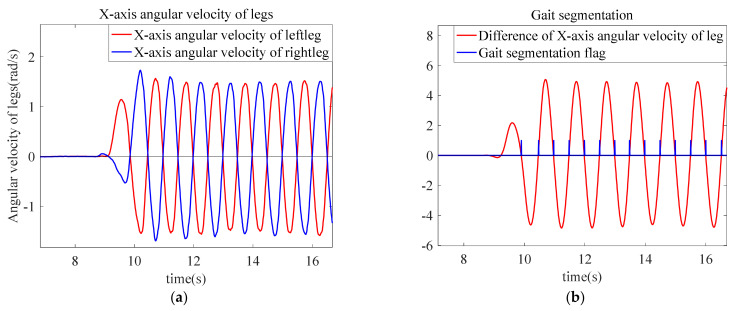
Digram of angular velocity of legs and gait segmentation: (**a**) *X*-axis angular velocity of legs; and (**b**) gait segmentation.

In Equation (10), $\omega_{ll}$ is the right angular velocity of the left leg, and $\omega_{rl}$ is the right angular velocity of the right leg. The leg angular velocity and gait segmentation are shown in Figure 4. In Figure 4a, the angular velocity of both legs is zero when a pedestrian is in a static state. When the pedestrian is moving, $\Delta \omega_{leg}$ is 0 only when the sensors of both legs swing to be perpendicular to the ground again. In Figure 4b, the red line is the difference between the right angular velocities of the two legs. When $\Delta \omega_{leg}$ is near the zero-crossing point, that means it has moved one step.

Through research, it is found that the Euclidean Distance of the acceleration and angular velocity of the legs can obviously distinguish the static and motion modes in static and small movement. The combined Euclidean Distance ρ of acceleration and angular velocity is calculated as

(11) $$\left\{\begin{array}{c} \rho =\sqrt{\rho_{1}\cdot \rho_{1}^{T}}+\sqrt{\rho_{2}\cdot \rho_{2}^{T}}\\ \rho_{1}=f_{ll}-f_{rl}\\ \rho_{2}=\omega_{ll}-\omega_{rl} \end{array}\right.$$

In Equation (11), f, ω are the acceleration and angular velocity information respectively, and ll,rl represent the left and right legs, respectively. According to ρ, the discrimination between stillness and motion is carried out, as shown in Equation (12). When it is less than the threshold $Th_{1}$, it’s in a static state, and the flag is set to 1. When it is greater than the threshold $Th_{1}$, it’s in a dynamic state, and the flag is set to 0.

(12) $$flag=\left\{\begin{array}{cc} 1 & \rho <Th_{1}\\ 0 & others \end{array}\right.$$

**Figure 5:**
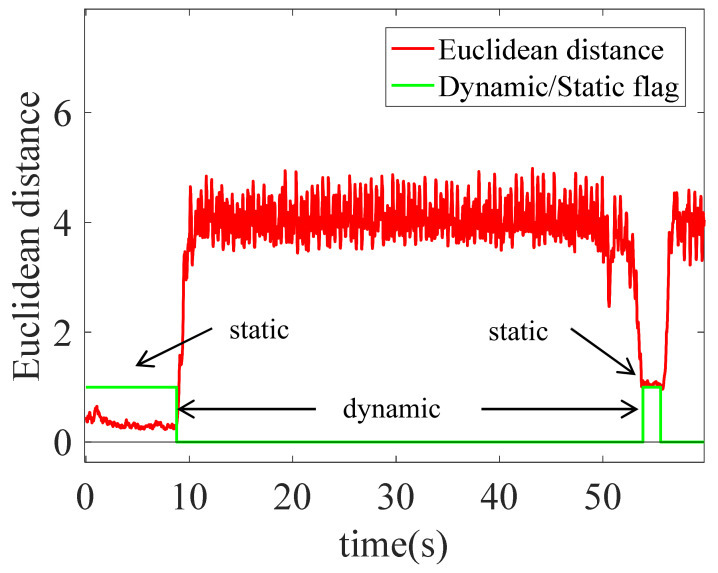
Recognition diagram of dynamic and static state.

The recognition effect of the static and dynamic states is shown in Figure 5.

#### 3.2.3. Recognition Algorithms for Dynamic State

The traditional classification methods are mainly classified by the threshold method. When there are many motion modes, a lot of logical judgments need to be made, and the robustness of the recognition algorithm is poor when the features change slightly in the process of moving. Therefore, this paper studies the method of pedestrian motion mode recognition based on machine learning. The commonly used classification methods of machine learning mainly include supervised learning, unsupervised learning, semi-supervised learning, and reinforcement learning. Supervised learning is to build a model from labeled training data and then input test set data into the model to predict its labels. The research on pedestrian motion modes recognition is applicable to supervised learning algorithms. Supervised learning algorithms mainly include KNN, SVM, DT, ANN, and other algorithms. Since the movement of pedestrians is a time series process with sequence characteristics, the movement data of one single moment cannot truly reflect the motion mode. Traditional machine learning methods are insensitive to sequence data. Recurrent Neural Network (RNN) has memory and has advantages in learning nonlinear features of data, but RNN has the problems of gradient disappearance and gradient explosion during backpropagation or training. In this paper, the LSTM algorithm is selected for motion mode recognition, which is a special RNN algorithm. LSTM inherits most of the features of the RNN model, while solving the gradient problem of RNN. LSTM can learn the time series rules of data more effectively, enhance the time dependence between data features, and has a certain memory ability for sequence data.

For the training set, the original data is preprocessed, then the classification eigenvalues are calculated, and the eigenvalues are input into the LSTM network to generate the classification network. For the test set, the original data are similarly processed, and the eigenvalues are calculated. Then the eigenvalues are input into the trained network to obtain the motion modes.

For the four common pedestrian motion modes of indoor walking, running, going upstairs, and going downstairs, firstly extract the eigenvalues to segment each step of the motion. The traditional method is to extract eigenvalues from the data in each step in the form of a sliding window. Usually, the recognition performance is improved by increasing the window length. However, there is a critical point in the window length. After the critical point is exceeded, the operation speed becomes slower, and the recognition accuracy does not improve much. For pedestrian motion mode recognition based on machine learning, the key technology is to find the eigenvalues with obvious features.

In this paper, the time-domain eigenvalues, such as mean value and mean square deviation, are extracted from the IMU data of two legs, and the calculated eigenvalues are coupled to generate new eigenvalues. The specific formula is as follows:

The mean value calculation expression is as Equation (13).

(13) $$\bar{x}=\frac{1}{N}\sum_{i=1}^{N}x_{i}$$

In the equation, N is the number of samples, and $x_{i}$ is the sample value.

The mean square error is calculated as Equation (14).

(14) $$\sigma =\sqrt{\frac{1}{\left(N-1\right)}\sum_{i=1}^{N}(x_{i}-\bar{x}{)}^{2}}$$

The mean square error is calculated as Equation (15).

(15) $$norm=\sqrt{\overset{N}{\underset{i=1}{\sum }}x_{i}^{2}}$$

The calculation formula of kurtosis is shown in Equation (16).

(16) $$Kurtosis=\frac{\sum_{i=1}^{N}{\left(x_{i}-\bar{x}\right)}^{4}}{\left(N-1\right)\cdot \sigma^{4}}-3$$

The calculation formula of skewness is shown in Equation (17).

(17) $$SK=\frac{\sum_{i=1}^{N}{\left(x_{i}-\bar{x}\right)}^{3}}{\left(N-1\right)\cdot \sigma^{3}}$$

The product of maximum and minimum values of forward acceleration of leg is calculated as Equation (18).

(18) $$Para_{1}=Acc_{y\max}\cdot Acc_{y\min}$$

The product of maximum and minimum values of right angular velocity of one leg is calculated as Equation (19).

(19) $$Para_{2}=\omega_{x\max}\cdot \omega_{x\min}$$

There are also the maximum leg forward acceleration $Acc_{y\max}$, and the maximum leg right angular velocity $\omega_{x\max}$.

## 4. Integrated Navigation Optimization Algorithm Based on ZUPT/Waist/Foot Heading Change

### 4.1. Navigation State and Measurement Modeling Based on ZUPT

The position, velocity, and attitude calculated by the pure SINS recursive algorithm will diverge with time, so other auxiliary methods need to be introduced to suppress the accumulation of errors. In this paper, the ZUPT method and the combination of waist/foot heading changes are used to periodically correct the navigation error, which can effectively improve the accuracy of the navigation system. 

**Figure 6:**
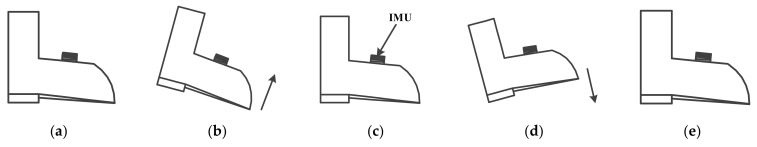
Decomposition diagram of foot motion: (**a**) static; (**b**) heel off the ground; (**c**) swing in the air; (**d**)toe to the ground; and (**e**) static.

During the movement of pedestrians, the motion state of foot can be roughly divided into the following four stages: static, heel off the ground, swing in the air, and toe to the ground. When the toes are off the ground, the heel has an obvious rotation process; After the foot lift, the foot swings in the air, and the body moves forward; After the leg swing is completed, it enters the heel landing stage, and then the forefoot touches the ground, and the acceleration value has a small amplitude pulse concussion. When entering the zero-velocity stage, the velocity is close to zero, and the sum of the acceleration modulus values is approximately the gravitational acceleration. The process of foot movement is shown in Figure 6. Through experimental research, the inertial sensor is installed on the instep, which can most sensitively sense the movement changes of the foot, and can reflect the movement process of the foot more realistically.

The 18-dimensional linear Kalman filter state quantity is constructed, as shown in equation (20). $\varphi_{e}$, $\varphi_{n}$, $\varphi_{u}$ are the platform angle error; $\delta V_{e}$, $\delta V_{n}$, $\delta V_{u}$ are the velocity error; δL, δλ, δh are the position error; $\varepsilon_{bx}$, $\varepsilon_{by}$, $\varepsilon_{bz}$ are the gyro random constant; $\varepsilon_{rx}$, $\varepsilon_{ry}$, $\varepsilon_{rz}$ are the gyro first-order Markov process random error; And $\nabla_{rx}$, $\nabla_{ry}$, $\nabla_{rz}$ are the accelerometer first-order Markov process random error.

(20) $$\begin{array}{l} X=\left[\begin{array}{cccccc} \varphi_{e} & \varphi_{n} & \varphi_{u} & \delta V_{e} & \delta V_{n} & \delta V_{u} \end{array}\right.\\ \begin{array}{cccccc} \delta L & \delta \lambda & \delta h & \varepsilon_{bx} & \varepsilon_{by} & \varepsilon_{bz} \end{array}\\ {\left.\begin{array}{cccccc} \varepsilon_{rx} & \varepsilon_{ry} & \varepsilon_{rz} & \nabla_{rx} & \nabla_{ry} & \nabla_{rz} \end{array}\right]}^{T} \end{array}$$

The error state equation of the established system is shown in Equation (21).

(21) $$\dot{X}{\left(t\right)}_{18\times 1}=A{\left(t\right)}_{18\times 18}X(t)+G{\left(t\right)}_{18\times 9}W{\left(t\right)}_{9\times 1}$$

Among them, A is the system matrix, X is the system state vector, G is the system noise matrix, and W is the system noise.

Based on the inertial/zero-velocity integrated navigation system, the velocity $v_{INS}$ and position $p_{INS}$ of the inertial navigation output at the current time and the corresponding velocity $v_{Last}$ and position $p_{Last}$ at the previous sampling time are measured by the difference as quantity. In the zero-velocity interval, Equation (22) is theoretically established.

(22) $$\begin{array}{l} \Delta v=v_{INS}-v_{Last}=0\\ \Delta p=p_{INS}-p_{Last}=0 \end{array}$$

When the zero-velocity interval is detected, $Z_{ZUPT}(t)$ is the measurement information, and the measurement equation is shown in Equation (23).

(23) $$Z_{ZUPT}(t)=\left[\begin{array}{c} v_{eINS}-v_{eLast}\\ v_{nINS}-v_{nLast}\\ v_{uINS}-v_{uLast}\\ (L_{INS}-L_{Last})R_{n}\\ (\lambda_{INS}-\lambda_{Last})R_{e}\cos L\\ h_{INS}-h_{Last} \end{array}\right]=H_{ZUPT}\left(t\right)X\left(t\right)+N_{ZUPT}\left(t\right)$$

In Equation (23), H is measurement matrix, X is the state vector, N is measurement noise, and *e*, *n* and *u*, respectively, represent east, north and universe direction. $v_{eLast}$, $v_{nLast}$, $v_{uLast}$ are zero-velocity, namely 0, 0 and 0. $L_{Last}$, $\lambda_{Last}$, $h_{Last}$ are the position information solved by inertial navigation, $L_{Last}$, $\lambda_{Last}$, $h_{Last}$ are the position information solved by inertial navigation at the last moment. $R_{n}$, $R_{e}$, respectively, represent meridian and unitary radius, and L is latitude.

### 4.2. Measurement Modeling Based on Waist/Foot Heading Changes

**Figure 7:**
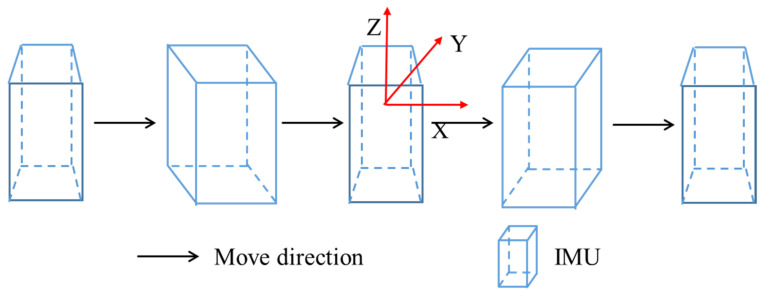
Decomposition diagram of waist motion.

The waist of pedestrians also changes periodically when they move. The decomposition diagram of waist motion is shown in Figure 7. According to the motion characteristics of waist angular velocity, it is found that the *z*-axis acceleration and *z*-axis angular velocity change the most, that is, the pedestrian waist heading has the most obvious change. The orientation of the IMU mounted on the waist returns to the original orientation twice for every two steps.

This paper studies pedestrian navigation based only on its own inertial sensors. The position and velocity errors can be corrected by relying on the ZUPT algorithm, but there is no external observable information to correct the pedestrian’s heading. When a person is moving, heading is calculated according to the pure inertial data of waist and foot. 

**Figure 8:**
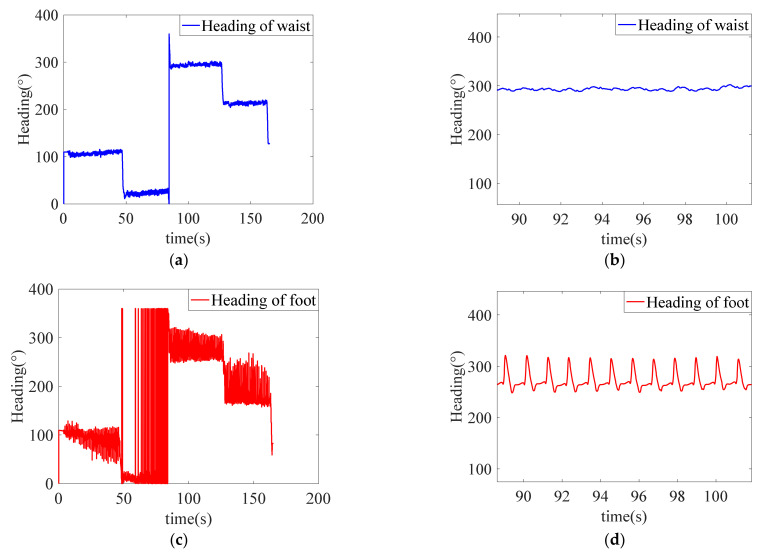
Comparison figures of heading of waist between heading of foot: (**a**) heading of waist; (**b**) enlarged view of heading of waist; (**c**) heading of foot; and (**d**) enlarged view of heading of foot.

Figure 8 are the comparison diagrams of heading of waist and heading of foot when walking a closed rectangular path. In the figures, for the convenience of observation, the initial heading of waist and foot are given the same value during parameter initialization. Figure 8 reflects the fact that the heading of waist is relatively more stable than foot. Figure 8c does not present a straight line as in Figure 8a due to foot swinging in the air. It can be seen from the enlarged detail of Figure 8d that when the foot is in the zero-speed interval, the heading of foot tends to be stable.

The movement of waist is smoother than that of foot, and the error of heading is smaller. However, in the actual moving process, the movement directions of waist and foot are not completely consistent, so heading of waist cannot be directly used as an observation. Theoretically, the variation of heading of waist $\Delta \psi_{waist}$ and heading of foot $\Delta \psi_{foot}$ should both be 0° for each step of walking, and the difference Δψ between $\Delta \psi_{waist}$ and $\Delta \psi_{foot}$ can be constructed as virtual observation. The observation information is shown in Equation (24).

(24) $$\begin{array}{l} \Delta \psi =\Delta {\bar{\psi }}_{waist}-\Delta {\bar{\psi }}_{foot}\\ \Delta \psi_{waist}={\bar{\psi }}_{waist_{k}}-{\bar{\psi }}_{waist_{k-1}}\\ \Delta \psi_{foot}={\bar{\psi }}_{foot_{k}}-{\bar{\psi }}_{foot_{k-1}} \end{array}$$

In the equation, ${\bar{\psi }}_{waist_{k}}$ and ${\bar{\psi }}_{waist_{k-1}}$, respectively, represent the mean value of waist heading of the current step and previous step, and ${\bar{\psi }}_{foot_{k}}$ and ${\bar{\psi }}_{foot_{k-1}}$, respectively, represent the mean value of foot heading of the current step and previous step.

The measurement equation is shown in Equation (25).

(25) $$Z_{\psi }(t)=\left[\Delta {\bar{\psi }}_{waist}-\Delta {\bar{\psi }}_{foot}\right]=H_{\psi }\left(t\right)X\left(t\right)+N_{\psi }\left(t\right)$$

The state equation is shown in Equation (26).

(26) $$H_{\psi }\left(t\right)=-\left[\begin{array}{cccc} \frac{\sin\psi *\sin\theta }{\cos\theta } & \frac{\cos\psi *\sin\theta }{\cos\theta } & -1 & 0_{1\times 15} \end{array}\right]$$

where θ is the pitch angle.

To sum up, the measurement equation of the combined system is shown in the Equation (27).

(27) $$Z(t)=\left[\begin{array}{c} v_{eINS}-v_{eLast}\\ v_{nINS}-v_{nLast}\\ v_{uINS}-v_{uLast}\\ (L_{INS}-L_{Last})R_{n}\\ (\lambda_{INS}-\lambda_{Last})R_{e}\cos L\\ h_{INS}-h_{Last}\\ \Delta {\bar{\psi }}_{waist}-\Delta {\bar{\psi }}_{foot} \end{array}\right]=H\left(t\right)X\left(t\right)+N\left(t\right)=\left[\begin{array}{l} H_{zupt}(t)\\ H_{\psi }(t) \end{array}\right]X(t)+\left[\begin{array}{c} N_{zupt}(t)\\ N_{\psi }(t) \end{array}\right]$$

## 5. Experiment and Result Analysis

### 5.1. Experimental Conditions

**Figure 9:**
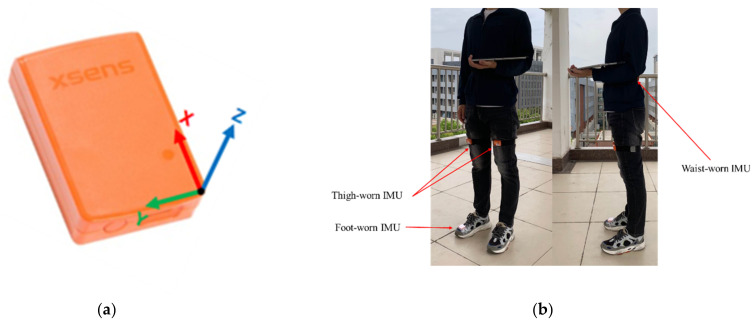
Appearance and installation configuration of MTW suit: (**a**) sensor appearance and object coordinate system; and (**b**) four-node inertial sensor network configuration.

The MTW inertial sensor suit from the Netherlands Xsens company is used in the experiment. The appearance and orientation of the sensor are shown in Figure 9a, and the wearing positions of the inertial sensor network are shown in Figure 9b. The sampling frequency of the accelerometer and gyroscope is 100 Hz, and the sampling frequency of the barometer is 50 Hz. The mass of the single sensor is 27 g and the size is 34.5 × 57.8 × 14.58 mm. The performance parameters of MTW are shown in Table 1.

**Figure 10:**
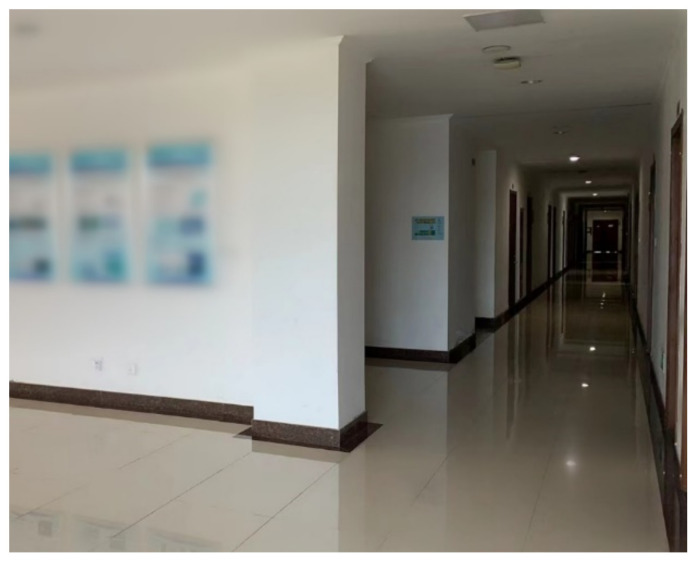
Indoor experimental scene.

Comprehensive tests are selected in indoor scenarios. The location is from the third floor to the fifth floor of the no.1 Building of the College of Automation Engineering. The indoor corridor scene is shown in Figure 10.

### 5.2. Analysis of Experimental Results

**Figure 11:**
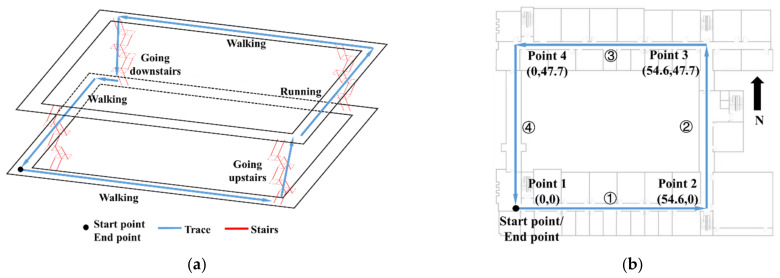
Trajectory of the experiment: (**a**) three-dimensional trajectory; and (**b**) two-dimensional trajectory projection.

In order to verify the validity of the motion mode recognition algorithm of the inertial sensor network and ZUPT + WF navigation algorithm proposed in this paper, two groups of repeated experiments were done. In this experiment, walking is the main motion mode. The three-dimensional path of the experiment is shown in Figure 11a. The start point starts from the third floor, then walks and goes upstairs to the fifth floor. Then run, walk, go downstairs to the third floor, and walk back to the origin. In order to evaluate the positioning error, the motion trajectory is projected onto a two-dimensional plane, and four reference points are set on the experimental path to calculate the error, as shown in Figure 11b. ①, ②, ③, and ④ represent the sequence of walking paths respectively. The coordinates of the reference point are (0, 0), (54.6, 0), (54.6, 47.7), (0, 47.7), and the unit is meters. The total length of the four straight lines of the corridor is 204.6 m. The vertical distance from the third floor to the fifth floor is 7.59 m.

#### 5.2.1. The Effect of Data Preprocessing

**Figure 12:**
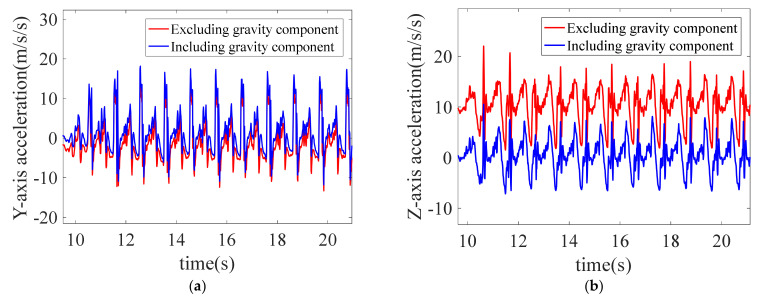
Comparison diagrams before and after deducting gravity component. (**a**) *Y*-axis acceleration before and after deducting gravity component; (**b**) *Z*-axis acceleration before and after deducting gravity component.

Every time the IMU is powered on to record motion data, keep the body still for 5 s to deduct the gravity component. The comparisons before and after deducting the gravity component by projection are shown in Figure 12.

**Figure 13:**
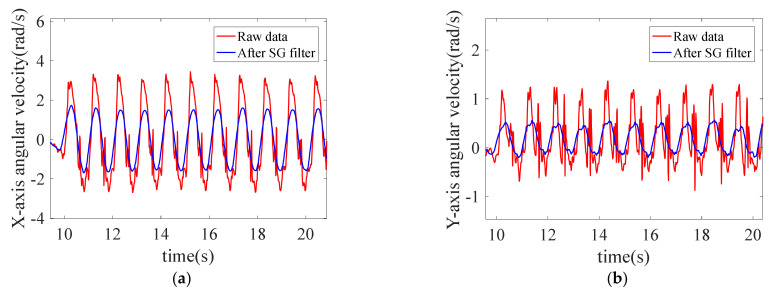
Comparison diagrams before and after SG filter: (**a**) *X*-axis angular velocity before and after SG filter; and (**b**) *Y*-axis angular velocity before and after SG filter.

SG filter is performed on the angular velocity of legs, and the comparison diagrams before and after filtering are shown in Figure 13. After filtering, the angular velocity data curve is smooth, with obvious periodicity and sinusoidal distribution.

#### 5.2.2. Algorithm Verification of Pedestrian Gait Segmentation and Motion Mode Recognition

**Figure 14:**
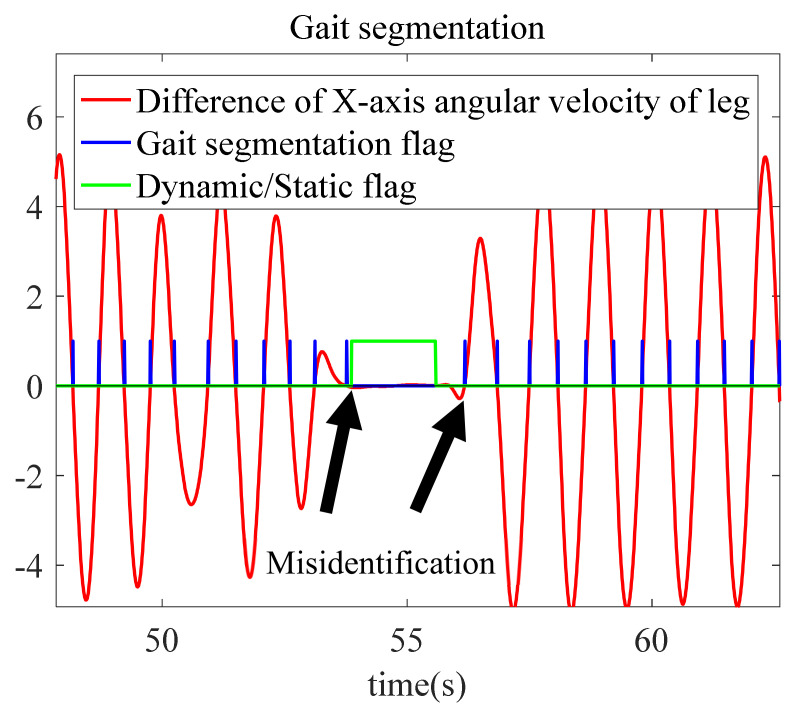
Diagram of gait segmentation.

In the two groups of experiments, the first experiment is taken as an example to illustrate in detail. In motion mode recognition, one single leg swing is taken as a step. According to the experimental data, the actual total number of movement steps is 434. Overall, the misrecognized gait segmentation is mainly in the start and stop phases of movement. In these two stages, the movement of a single leg is small, and the eigenvalues are relatively close, which is prone to misjudgment as shown in Figure 14.

After statistics, the gait segmentation recognition rates of the two groups of experiments are shown in Table 2.

**Figure 15:**
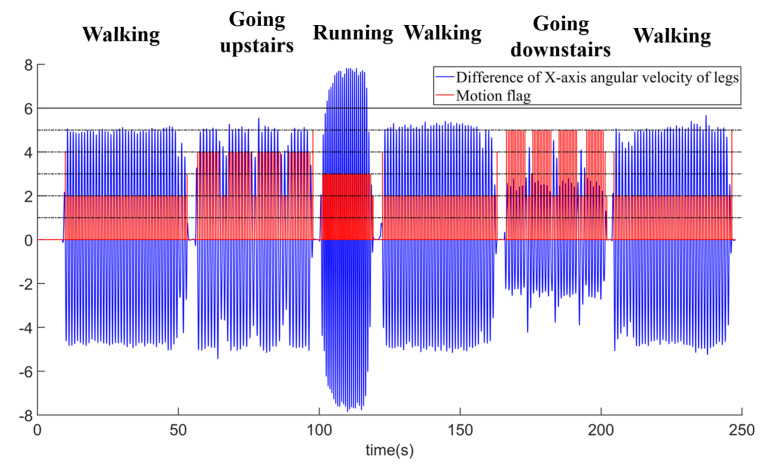
Recognition of multi-motion modes with LSTM for pedestrians.

Since the motion amplitude of one leg is not large and the eigenvalues are close, the motion modes are also easily confused at the start and stop stages. However, in any continuous motion mode, it is not easy to appear eigenvalues close to other motion modes, and the mode recognition rate is high in the motion process. In running mode, the pedestrian’s velocity gradually increases when he starts running, and slows down when he is about to stop running. Two steps before running and two steps after running can be considered as walking mode, so it is not included in error detection. For going upstairs from the third floor to the fifth floor and going downstairs from the fifth floor to the third floor, these two motion modes are performed continuously. In the process of going upstairs and going downstairs, it also moves when passing through the platform at the corner. The modes in continuous motion are modified by filtering and smoothing algorithms. Table 3 shows the comparative statistics of various accuracy rates of motion modes recognition. Figure 15 shows the recognition results of multi-motion modes of the human body based on the LSTM algorithm. KNN algorithm has the lowest recognition rate for going downstairs. DT algorithm is the best for walking, but poor for recognizing going downstairs; The overall accuracy of the SVM algorithm exceeds 90%; Overall, the LSTM algorithm has the highest accuracy. Among the misrecognized points, no running is misrecognized as other sports, nor other sports are misrecognized as running. Therefore, it does not affect the zero-velocity detection in the subsequent navigation algorithm research.

#### 5.2.3. ZUPT Algorithm and Verification

Based on the requirement of pedestrian navigation accuracy, it is necessary to accurately recognize the zero-velocity interval. Aiming at the problem of insufficient accuracy and weak robustness of recognizing zero-velocity detection with a single threshold value, a multi-threshold combinating zero-velocity detection method is constructed. In this method, discriminant eigenvalues are used for recognizing the zero-velocity interval with acceleration mean square error, acceleration modulus, angular velocity mean square error and angular velocity modulus in a sliding window. The specific sub-zero-velocity discrimination method is as follows:

(28) $$\left\{\begin{array}{c} a_{i}=\sqrt{f_{x}{}^{2}+f_{y}{}^{2}+f_{z}{}^{2}}\\ \sigma_{a}=\frac{1}{k+1}\sum_{i=n}^{n+k}{\left(a_{i}-\bar{a}\right)}^{2}\\ \lambda_{1}(k)=\left\{\begin{array}{ll} 1 & \;\;\;\sigma_{a}<\varepsilon_{a_{1}}\\ 0 & \;\;\;others \end{array}\right. \end{array}\right.$$

(29) $$a_{v}=\left(\frac{a_{l}\cdot a}{a\cdot a}\right)\cdot a$$

(30) $$a_{v}=\left(\frac{a_{l}\cdot a}{a\cdot a}\right)\cdot a$$

(31) $$a_{v}=\left(\frac{a_{l}\cdot a}{a\cdot a}\right)\cdot a$$

In Equations (28)–(31), k represents sliding window size, f represents the acceleration, ω represents angular velocity; *x*, *y*, and *z* represent the three axes of the accelerometer and gyroscope sensor; $\varepsilon_{a_{1}}$ and $\varepsilon_{a_{2}}$ represent the set acceleration mean square error threshold and acceleration modulus threshold, respectively. $\varepsilon_{\omega_{1}}$, $\varepsilon_{\omega_{2}}$, respectively, represent the set gyro mean square error threshold and gyro modulus threshold. The zero-velocity interval is detected by the combination of these four kinds of zero-velocity detection thresholds, and the detection accuracy is improved.

In view of the fast velocity in the running state, the zero-velocity interval is shorter than the other three motion modes, and the eigenvalues change greatly. The characteristics of smooth movement (walking, going upstairs, going downstairs) and non-smooth movement (running) are analyzed, and different zero-velocity detection methods are set up, respectively. The comprehensive judgment scheme of zero-velocity detection is shown in Equation (32), when the motion mode is walking, going upstairs, or going downstairs.

(32) $$Z_{1}(k)=\left\{\begin{array}{ll} 1 & \lambda_{1}(k)\&\&\lambda_{2}(k)\&\&\lambda_{3}(k)\&\&\lambda_{4}(k)\\ 0 & \;\;\;\;\;\;\;\;\;\;\;\;\;\;\;\;\;\;\;\;\;\;others \end{array}\right.$$

When the motion mode is running,

(33) $$Z_{2}(k)=\left\{\begin{array}{ll} 1 & \left(\lambda_{1}(k)||\lambda_{3}(k))\&\&(\lambda_{2}(k)||\lambda_{4}(k)\right)\\ 0 & \;\;\;\;\;\;\;\;\;\;\;\;\;\;\;\;\;\;\;\;\;others \end{array}\right.$$

**Figure 16:**
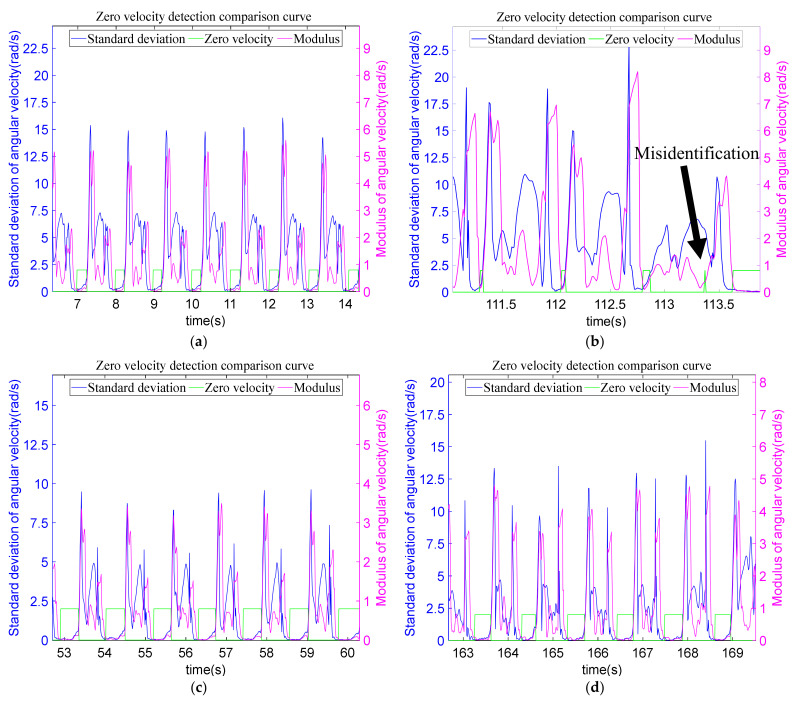
Detections of zero-velocity interval: (**a**) walking; (**b**) running; (**c**) going upstairs; and (**d**) going downstairs.

The detection figures of zero-velocity detection are shown in Figure 16. In order to facilitate viewing the zero-velocity interval in the figure, the value of the flag of the zero-velocity interval is temporarily increased here. The zero-velocity detection method studied in this paper can accurately recognize the zero-velocity interval and provide a basis for subsequent pedestrian navigation based on ZUPT.

The statistics of the number of movement steps for the two groups of comparative experiments are shown in Table 4. For the same experimenter, the stride and velocity of the movement are similar, so the number of steps in the two groups is almost the same. When making statistics, one step is taken as the number of swings of right foot, and a zero-velocity interval is between two steps. Compared with the number of steps in motion mode recognition, the data in the zero-velocity interval is reduced by half, and the recognition rates of the two groups in the zero-velocity interval are 99.5% and 100%, respectively.

#### 5.2.4. Verification of Combined Algorithm Based on Zero-Velocity/Waist Heading Change Constraints

In order to ensure the reliability of the experimental results, the coordinate points (0, 0), (54.6, 0), (54.6, 47.7), (0, 47.7) at the four corners of the experimental path are used as reference points to calculate the error between the position solved by the algorithm and the actual position. Root Mean Squared Error (RMSE) is used as the error evaluation method, and RMSE is calculated by Equation (34):

(34) $$RMSE=\sqrt{\frac{1}{n}\sum_{i=1}^{n}{\left(X_{obs,i}-X_{ref,i}\right)}^{2}}$$

Among them, n is 4, $X_{obs,i}$ is the estimated position of the algorithm, $X_{ref,i}$ is the reference point position, and RMSE is the final positioning error.

**Figure 17:**
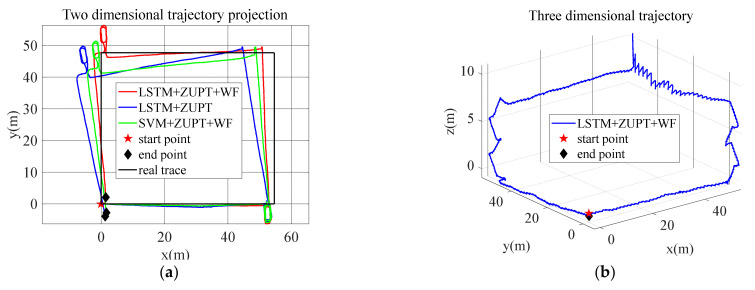
Pedestrian experimental trajectory: (**a**) two-dimensional trajectory projection; and (**b**) three-dimensional trajectory.

The LSTM + ZUPT + WF navigation algorithm proposed in this paper is compared with the traditional LSTM + ZUPT algorithm. The positioning results are shown in Figure 17, and the error comparison of the positioning results is shown in Table 5. In Figure 17a, the red line represents the proposed LSTM + ZUPT + WF algorithm, the blue line represents the traditional LSTM + ZUPT algorithm, and the green line represents the SVM + ZUPT + WF algorithm. It can be seen that the proposed LSTM + ZUPT + WF navigation algorithm has higher precision and lower RMSE value than the traditional LSTM + ZUPT algorithm and SVM + ZUPT + WF. In Figure 17b, the blue line represents the 3D trajectory of the LSTM + ZUPT + WF navigation algorithm.

## 6. Discussion

The purpose of this paper is to develop an autonomous multi-motion modes recognition and navigation optimization method suitable for indoor pedestrians. In this paper, the mode recognition algorithm based on gait segmentation is studied, which can achieve accurate segmentation of the four motion modes of walking, running, going upstairs, and going downstairs. According to the motion characteristics in each step of the four motion modes, the highly differentiated motion characteristic values are selected to train the machine learning network of LSTM for motion mode recognition. Because the traditional zero-velocity interval detection only studies the walking state, the zero-velocity interval judgment method is not applicable to multi-motion modes. Combined with the characteristic that the zero-velocity interval in the running state is shorter than the other three motion modes, on the basis of motion mode recognition, the motion modes are divided into two categories: walking, going upstairs, and going downstairs as one category, and running as the other category, a hierarchical zero-velocity interval recognition framework is constructed. For the traditional LSTM + ZUPT-based pedestrian navigation algorithm, since there is no external heading information correction, the moving heading will drift gradually over time. Therefore, a navigation method based on waist/foot heading information is studied, and a pedestrian navigation method based on LSTM + ZUPT + WF is proposed. The virtual observation is constructed by the difference between the pedestrian’s own waist heading and foot heading to correct the navigation heading. In order to verify the effectiveness of the method proposed in this paper, two sets of indoor comprehensive experiments are conducted. The experiments include four motion modes: walking, running, going upstairs, and going downstairs. The walking mode accounts for the highest proportion in the experiment. Through the experimental data, it is verified that the accuracy of the proposed gait segmentation algorithm exceeds 98.60%, the comprehensive recognition rate of the four motion modes is 96.77%, and the detection rate of the zero-velocity interval is more than 99.00%. Compared with the traditional LSTM + ZUPT navigation algorithm, The RMSE of the navigation algorithm based on LSTM + ZUPT + WF is reduced by 59.70%. For the horizontal projection trajectory of pedestrian movement, the difference between the start point and the end point is 2.59 m, and the error is 1.26% of the total distance. Reference [18] uses the SVM algorithm for recognition. Compared with SVM + ZUPT + WF, LSTM + ZUPT + WF has better classification accuracy and navigation accuracy than SVM + ZUPT + WF. Without relying on external sensors, the proposed method can provide high-precision navigation and positioning information for indoor pedestrians with multi-motion modes, which is of great significance for the practical application of pedestrian inertial navigation.

Although the algorithm proposed in this paper can provide high-precision navigation and positioning information, there are still some limitations. First, the method does not use external sensor assistance, and the initial heading of the pedestrian needs to be set manually; Secondly, only four common motion modes have been studied so far, and special indoor tasks may also include jumping, retreating, sideways movement, or even crawling and climbing, etc. Further research can be carried out according to actual needs in the future; Then, if the heading cannot be corrected for a longer period of movement, UWB anchors can be deployed in advance at special positions during the movement, such as a corner of the corridor; Finally, only single pedestrian navigation technology is studied. If special tasks are performed indoors, indoor features can be fully utilized, or information can be shared among task team members to further improve the navigation and positioning accuracy. Only single pedestrian navigation technology is studied. If indoor special tasks are carried out, indoor features can be fully utilized, or information can be shared among task group members to further improve navigation and positioning accuracy. The algorithm proposed in this paper has high accuracy in practical navigation and positioning and has good research value. It can be used in multi-person cooperative navigation research in the future.

## Figures and Tables

**Table 1:** Parameters of MTW.

/	Full Scale	Bias Stability
Acceleration	±160 m/s^2^	/
Angular velocity	±1200 deg/s	20 deg/h
Pressure	300–1100 mBar	100 Pa/year

**Table 2:** Recognition rate of gait segmentation.

/	Number of Real Steps	Number of Steps Detected	Accuracy
Experiment 1	434	437	99.31%
Experiment 2	430	436	98.60%

**Table 3:** Recognition results of multi-motion modes.

Motion Mode	Real Steps	Recognition Rate with KNN (%)	Recognition Rate with DT (%)	Recognition Rate with SVM (%)	Recognized Steps with LSTM	Recognition Rate with LSTM (%)
Walking	270	95.56	98.52	93.33	260	96.30
Running	52	96.15	98.08	96.15	52	100.00
Going upstairs	56	94.23	91.07	92.86	56	100.00
Going downstairs	56	82.14	26.79	89.29	52	92.86
Subtotal	434	93.78	88.25	93.55	420	96.77

**Table 4:** Detection of zero-velocity interval.

/	Motion Modes	Real Steps	Detecting Steps	Accuracy
Experiment 1	Walking + going upstairs + going downstairs	182	183	99.85%
	Running	25	25	
Experiment 2	Walking + going upstairs + going downstairs	182	182	100%
	Running	25	25	

**Table 5:** Error comparison of positioning results.

/	Distance between the Start Point and End Point with LSTM + ZUPT (Meter)	RMSE with LSTM + ZUPT (Meter)	Distance between the Start Point and End Point with SVM + ZUPT + WF (Meter)	RMSE with SVM + ZUPT + WF (Meter)	Distance between the Start Point and End Point with LSTM + ZUPT + WF (Meter)	RMSE with LSTM + ZUPT + WF (Meter)
Experiment 1	4.21	7.71	3.21	4.88	2.59	2.67
Experiment 2	4.89	10.23	4.72	5.09	2.76	4.12
